# Treatment of Fistula in Ano*Read before the Northern Tri-States Medical Association, July 17, 1894.

**Published:** 1894-10

**Authors:** George J. Cook

**Affiliations:** 118 West Ohio street, Indianapolis, Ind.


### Selections and Abstracts.

---

TREATMENT OF FISTULA IN ANO.*—The ingenuity of surgeons has been exercised in all ages to cure fistula without the use of the knife, because of the natural fear of this instrument, especially in connection with this disease. Over two centuries ago the royal patient of Felix spent a year trying all manner of remedies, from mineral waters to the plaster of Madame de la Daubiere, for the relief of his fistula, before he would submit to the treatment that

*Read before the Northern Tri-States Medical Association, July 17, 1894.

finally cured him. And human nature is the same to-day; the surgeon's knife is the last resort, though the patient may have used remedies much more painful. And the decision of Bessieres, after examining the fistula of Louis, that "all the remedies in the world are of no account without an operation," is applicable to the great majority of anal fistulas to-day.

The success of Felix in his operation with the knife on Louis XIV., in 1686, established this operation for the cure of fistula, and it has stood ever since. Cutting is the only treatment for fistula which a surgeon can use and positively promise a cure. Every other means is uncertain. It is true that some fistulas have been cured by passing a probe through the tract, the irritation being sufficient to excite the healing process. Again many have been cured by cauterization of the tract, or the application of stimulating medicines; but no surgeon can truthfully promise positively to cure a fistula by the application of medicines alone, no difference how simple or recent it may be. I do not know of any new method for treating a fistula. All the methods adopted now were used centuries ago. The technique may be different, especially since the advent of antiseptic surgery, and the results of treatment are certainly much more favorable.

The ligature is as old as surgical history, and in the time of Louis XIV. one Lemoyne grew rich treating fistulas by means of caustics. The method of treating by caustics or stimulants at present is about as follows: The patient having been prepared by thoroughly clearing the bowels, the fistulous tract is cleaned by injecting peroxide of hydrogen*; then the caustic is injected, which is usually carbolic acid from 50 per cent. solution to pure acid, or nitrate of silver from 30 to 60 grains to an ounce of water. After getting the full effect of the caustic on the walls of the fistula, then inject oil of eucalyptus, pure or mixed with equal quantity of oil of sweet almonds, or a solution or mixture of iodoform; then if the fistula is located so that pressure can be made, apply a firm pad of cotton, and hold it tight by a T bandage and keep the patient quiet for a day or two. This treatment is thoroughly antiseptic and will cure sometimes; I have had success with this treatment when I little expected it. But the most that can be promised the patient is that it may cure, and if it does not no harm will be done, and other means can be adopted later if necessary. This method is applicable only to simple fistulas recently formed, or those in which there is not much induration around the tract, the object of the application being to promote granulation and remove the inflammatory exudate. The same object may be attained by scarification of

*Marchand's is the standard preparation.—ED.

the walls of the tract. For this purpose an instrument has been devised by Dr. Mathews, similar to the urethrotome, but with two knives. The instrument is passed through the tract as a probe, then the knives are exposed and the instrument withdrawn, making two incisions through the opposite sides of the fistula along its entire extent. If the fistula is branched, it will be a waste of time to try either injection or scarification. The ligature is one method of cutting open a fistula. A number of years ago Dittel brought the elastic ligature into prominence, and later Allingham made it quite popular for a time, but this at best is slow and painful, and now it has almost passed out of use. Some surgeons have used the cautery knife to open the fistula, and at the same time cauterize the tract. I cannot see any advantage in this; the edges cannot be trimmed as nicely, there is likely to be more irritation than we wish, and the healing process will certainly not be more rapid than when we use simply the knife.

The electro-cautery wire has also been used to open the tract, but the same objection would apply to this as to the thermo-cautery knife. The best and only certain treatment for a fistula, and applicable alike to all forms, is by means of the surgeon's knife and scissors. The usual process is familiar to all, and I will not take time repeating it.

During the past two years I have used the modification of this method, practiced several years ago by Dr. Frederick Lange, which consists of opening the fistulous tract and cutting out the indurated tissue surrounding it, thus converting it into a simple healthy wound, then bringing together the raw surfaces with sutures with the object of securing immediate union. Antiseptic surgery makes this possible. Cutting the fistula out is a very old practice, but before the date of antisepsis no attempt was made to have immediate union; it was rare indeed that such results could then have been accomplished.

I have had very favorable results from this operation, and now I adopt it in all cases where the wound can be closed after the indurated tissue is removed. If the fistula is of such character that the opposite surface could not be brought together and properly adjusted after removing the induration, no attempt should be made to remove it, but thorough scarification should be done with the knife after opening the fistula, and the tract be allowed to close by granulation. Formerly the difficulty in this operation was to secure union of the deep part; often the surface would heal, leaving a fistula at the bottom greater in extent than the original one. To remedy this Lange used two rows of sutures—a deep row of buried sutures to close the bottom of the wound, and a superficial row to close the surface.

My method of operating is as follows: The patient is prepared in the usual way for such operations. After the sphincter is thoroughly stretched, the rectum is washed out carefully with bichloride solution 1 to 4,000, then the fistula is split open; for this a pair of strong, straight scissors will be found better than a knife. Then remove the indurated tissue, which should be done thoroughly, taking care to cut out clean both ends of the fistula, not leaving any pale unhealthy skin or mucous membrane at these points. Search carefully for branch channels, and when found treat them in the same way, using the bichloride solution freely during all this time. To close the wound use catgut, and only a single row of sutures.

With a curved needle pass each suture entirely beneath and around the wound, not allowing the suture to come through or appear in the bottom of the wound. They should be placed close together, and great care used in placing the upper ones in the mucous membrane, so as to thoroughly close that end of the wound. When the sphincter is cut care is necessary to have the ends approximated, and secure a good muscle afterward. When all the sutures are passed wash out the wound again before tying; then when the wound is closed cover the parts with aristol, and keep the patient in bed as quiet as possible for several days; keep the bowels quiet with opium for four or five days, then wash them out by injection of hot water. By placing the sutures in this way the bottom of the wound can be brought closely together, and as well or better than by a row of buried sutures. Septic matter cannot enter the wound along the sutures, because they do not touch the wound surface. I have not failed yet in a single instance to have the bottom of the wound unite. In some of the cases one or two of the stitches cut through the skin and allowed a slight separation, but it was only of the skin and healed promptly. I have closed some very deep fistulas in this way with perfect results. In some cases where the main fistula could not be closed, I closed the branches or a part of the main tract, and lessened very much the part to close by granulation. Where immediate union is secured in this way the advantages are very great:

*First*, There is no gap left in the sphincter muscle, which is of special importance in females, if the muscle has to be cut anywhere in its anterior part, or in any case where the muscle has to be cut more than once.

*Second*, The patient is well in a week.—GEORGE J. COOK, M.D., 118 West Ohio street, Indianapolis, Ind., in *Mathews' Medical Quarterly*.

